# Utilization patterns of traditional medicine in Taiwan and South Korea by using national health insurance data in 2011

**DOI:** 10.1371/journal.pone.0208569

**Published:** 2018-12-27

**Authors:** Ching-Wen Huang, I-Hsuan Hwang, Ye-seul Lee, Shinn-Jang Hwang, Seong-Gyu Ko, Fang-Pey Chen, Bo-Hyoung Jang

**Affiliations:** 1 Department of Preventive Medicine, College of Korean Medicine, Kyung Hee University, Seoul, Korea; 2 Department of Science in Korean Medicine, Graduate School, Kyung Hee University, 26 Kyungheedae-ro, Dongdaemun-gu, Seoul, South Korea; 3 Center for Quality Control, Cheng Hsin General Hospital, Taipei, Taiwan; 4 Department of Anatomy and Acupoint, College of Korean Medicine, Gachon University, Seongnam, South Korea; 5 Department of Family Medicine, Taipei Veterans General Hospital, Taipei, Taiwan; 6 Department of Family Medicine, National Yang-Ming University School of Medicine, Taipei, Taiwan; 7 Center for Traditional Medicine, Taipei Veterans General Hospital, Taipei, Taiwan; 8 Institute of Traditional Medicine, National Yang-Ming University School of Medicine, Taipei, Taiwan; Institute of Health Economics and Health Care, GERMANY

## Abstract

**Background:**

The growing popularity of traditional medicine (TM) is reflected in the increasing trend for its use worldwide. Many people are turning to use TM as a complementary or integrative treatment. The aim of this study is to present the first nationwide report describing the use of TM in two countries (South Korea and Taiwan).

**Materials and methods:**

To present the TM utilization patterns between South Korea and Taiwan, we analyzed data from the National Health Insurance cohorts in each country, each of which has approximately one million inhabitants.

**Results:**

In total, 261,478 (25.5%) of 1,025,340 people in South Korea and 260,529 (26.8%) of 970,866 people in Taiwan used TM services at least once under the National Health Insurance in 2011. Using multivariable logistic regression, TM users in South Korea were significantly more likely to be female, 61–80 years of age and individuals with a high income, and those in Taiwan were significantly more likely to be female, 21–40 years of age and individuals with a middle income. The two countries showed similar utilization patterns in visit seasons. People visited TM clinics more frequently than TM hospitals in both countries. The most common TM treatment in South Korea was acupuncture, whereas in Taiwan, various powdered Chinese herbal preparations were the most commonly used treatment. The most common diseases for people seeking TM services were musculoskeletal system and connective tissue diseases in South Korea and Symptoms, signs, and ill-defined conditions in Taiwan.

**Conclusion:**

According to the National Health Insurance database, about one fourth of the NHI beneficiaries of South Korea and Taiwan had TM use in 2011. Different TM utilization patterns existed between South Korea and Taiwan, which might be due to the differences in insurance coverage between the two countries.

## Introduction

Traditional medicine (TM) is a medical field that maintains health, prevent and treat illnesses based on its theories [1]. The growing popularity of TM is reflected in the increasing trend for its use worldwide [2]. Furthermore, the role of TM in Asian countries such as South Korea, Taiwan, Japan, and China is not only limited to an alternative therapy but is extended to a primary treatment for some diseases [1].

The health care systems and the National Health Insurance (NHI) systems are similar in Taiwan and South Korea. In Taiwan, most of the medical expenditures of residents have been reimbursed by the NHI since 1995 [3]; a year later, health coverage for TM by the NHI was also included [4]. At the end of June, 2014, over 99.9% of inhabitants in Taiwan were covered in the NHI [5]. In Taiwan, the TM services covered by the NHI included powdered Chinese herbal preparations, acupuncture and moxibustion, and manipulative therapy [4]. Specifically, 337 types of Chinese herbal formula and over 500 types of Chinese single herb preparations are covered by the Taiwan NHI. These TM services accounted for approximately 3.1% of the total medical expenditures in the Taiwan NHI program in 2011.

In South Korea, universal health coverage was achieved in 1987, and TM has been included in the health coverage by the NHI since the same year. At the end of 2012, 49,622,000 individuals (97.1% of the population) were enrolled in the NHI system. The TM services covered by NHI in South Korea include acupuncture, moxibustion, cupping therapy and powdered Korean herbal preparations. However, only 58 types of herbal formula and 68 single herb preparations are included in the NHI coverage. These TM services accounted for approximately 3.9% of the total medical expenditures in the Korean NHI program in 2013 [6].

Previous studies on the patterns of TM healthcare utilization were primarily characterized by small sample sizes or were based on sampling surveys. Since 1997, the Taiwan National Health Insurance Research Database (TNHIRD) from the National Health Insurance Research Institute has been released for academic research use. The TNHIRD includes more than 26 files, such as the Registry for Beneficiaries, Ambulatory Care Expenditures by Visit and Details of Ambulatory Care Orders. On the other hand, South Korea has also released the Korea National Health Information Database (KNHID) which contains the records of all NHI data from the Korea National Health Insurance service (KNHIS). In addition, the KNHIS has been releasing Korea National Health Insurance-National Sample Cohort (KNHIS-NSC) data since 2014, which include qualification data, medical treatment databases, health examination databases and clinic databases. Other countries, such as Japan and the Scandinavian countries, have similar NHI databases; however, their sizes are small, and the cross-linkage among the registries is not sufficient in Japan and Scandinavia databases [7–10].

To date, Taiwan and South Korea are the only two countries that have released detailed NHI data to researchers. The use of largely complete NHI databases allows us to reduce concerns about selection effects. Larger sample sizes also give more precision for estimates. However, only a few studies are available to describe the TM utilization patterns between these two countries. Therefore, this study employed national population-based databases from both Taiwan and Korea to describe the pattern of TM utilization between the two countries. The aim of this study is to present the patterns of TM treatment, diseases treated with TM use and the reimbursement system of TM of the two countries, respectively.

## Materials and methods

### Data sources

The first database used in this study is the Taiwan Longitudinal Health Insurance Database (LHID2000) released by the TNHIRD. A total of one million individuals were randomly selected from the Registry for beneficiaries in 2000, and all their claims information from 1996 to 2013 were recorded in the data labelled LHID2000. These are stratified sampling cohort data which represent Taiwanese NHI beneficiaries nationwide (23.75 million). The age, sex and economic level data distributions showed no significant differences between the sampling data and the entire TNHIRD. In this study, we used the Registry for Beneficiaries (ID), Registry for Contracted Medical Facilities (HOSB), ambulatory care expenditures by visits (CD), and details of ambulatory care orders (OO) files from LHID2000. We extracted the reimbursement claimed records in 2011 in this analysis.

The other data used in this study were retrieved from the National Sample Cohort 2002–2013 (NHIS-NSC 2002–2013) of the KNHIS in South Korea. This dataset also included approximately one million inhabitants who were randomly sampled in 2002, with no significant differences in age, sex and economic level compared to the entire population. In this study, the Qualification data, T120t data (Statement file of western medicine), T220t data (Statement file of dental and oriental medicine), T130t data (Details of treatment file of western medicine) and T230t data (Details of treatment file of dental and oriental medicine) in the medical treatment database and Clinic database in the National Health Insurance Service–National Sample Cohort 2002–2013 (NHIS-NSC 2002–2013) were used. We extracted the reimbursement claimed records in 2011 in this study.

All data used in this study were anonymized to protect patient privacy prior to release from the National Health Institute Research Institutes. This study received approval and certification from the Institutional Review Board (IRB) of Kyung Hee University in South Korea (Approval # KHSIRB-15-057 (EA) and the IRB of National Yang-Ming University in Taiwan (YM105098E).

### Study process

**Fig 1:**
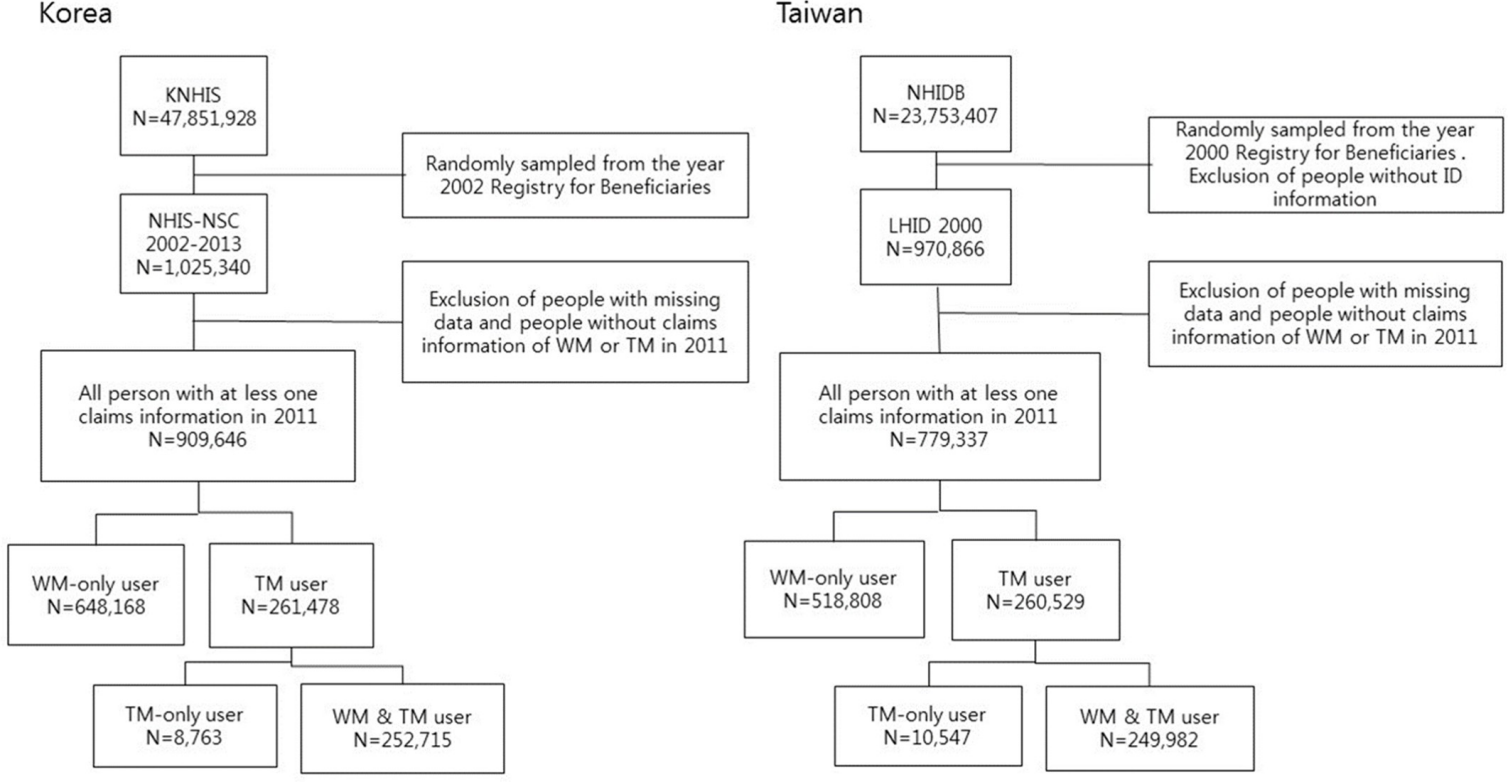
Flow recruitment chart of subjects from the National Insurance systems in South Korean and Taiwan.

The dataset from Taiwan (LHID2000) in this study was a randomly selected cohort from the Registry for Beneficiaries in 2000. Similarly, the dataset from South Korea (NHIS-NSC 2002–2013) used in this study was a randomly selected cohort from the Registry for Beneficiaries in 2002. The extracted reimbursement claimed records of the two countries showed that there were 1,025,340 and 970,866 beneficiaries in South Korea and Taiwan, respectively. The age and sex distribution of people selected from the 2011 ID files were not significantly different from the National Statistics of both countries. After excluding the records of the patients who visited dental clinics and the patients who did not use medical service covered by NHI, there were 909,646 and 779,337 people who had at least one claim data point for WM or TM in South Korea and Taiwan, respectively (Fig 1).

The subjects were categorized into three groups as follows: 1) WM-only users who only visited the WM institutes at least once in 2011, 2) TM-only users who sought only the TM service at least once in 2011, and 3) others who visited both WM institutes and TM institutes, and were categorized as WM & TM users. The TM users in this study included both TM-only users and WM & TM users, which encompassed all patients who received TM services at least once regardless of whether or not they used WM (Fig 1).

Demographic characteristics, including sex, age, and economic level, were analyzed for each group. Economic level was categorized into four groups according to monthly income (10 levels) in Taiwan and the level of insurance (10 levels) in South Korea: low economic level (a monthly income lower than 17,880 new Taiwan dollars [NTD] in Taiwan and an insurance amount lower than 23,980 Korean Won in South Korea), low-middle economic level (a monthly income ranging from 17,880 to 28,800 NTD in Taiwan and an insurance amount ranging from 23,980 to 28,590 Korean Won in South Korea), middle economic level (a monthly income ranging from 28,800 to 45,800 NTD in Taiwan and an insurance insured amount ranging from 28,590 to 41,730 Korean Won in South Korea), and high economic level (a monthly income over 45,800 NTD in Taiwan and an insurance amount over 41,730 Korean Won in South Korea).

Disease diagnoses were categorized according to the International Classification of Diseases, Ninth Revision, Clinical Modification (ICD-9-CM) in Taiwan and the ICD-10 in South Korea.

### Statistical analysis

Data in the text and tables were expressed as the frequency, mean ± standard deviation, median and range. Descriptive statistics were used in the patients’ demographic characteristics, WM & TM use, and use of medical services. This study used the Chi-square test to examine the relationship between each variable and the differences among the WM-only, TM-only, and WM & TM users. To analyze the predictive variables of TM users, we used univariable and multivariable logistic regression, with TM use as the dependent variable and age, sex, and economic level as independent variables. Multivariable logistic regression models included sex, age, and economic level. All data were analyzed using SAS version 9.3 (TS1M2). Two tailed tests were used, and a *P*-value < 0.05 was considered to be significant.

## Results

### Demographic characteristics of WM-only users, TM-only users and those who used both WM and TM

In total, 261,478 (25.5%) of the 1,025,340 people in South Korea and 260,529 (26.8%) of the 970,866 people in Taiwan used TM services at least once under the NHI in 2011. Table 1 shows the characteristics of the WM-only users, TM-only users, and WM & TM users in South Korea and Taiwan. In both countries, significant differences existed in sex, age and economic level between the WM-only users, TM-only users, and WM & TM users (all *P* < 0.001). Univariable and multivariable logistic regression analyses of the TM users (include both TM-only and WM & TM users) are listed in Table 2. Using univariable logistic regression, being female was associated with higher odds of TM use. A pattern of increasing odds for TM use with greater age, peaking at 61–80, but then declining for those aged over 80 in South Korea. There was evidence of decreasing use for high economic level in South Korea. On the other hand, being female was also associated with higher odds of TM use in Taiwan. A pattern of decreasing odds for TM use was seen with greater age since 21–40 onwards. There was evidence of increasing use for high economic level in Taiwan. Using multivariable logistic regression (adjusted for sex, age and economic level), the pattern was the same as univariable logistic regression in age and sex but there was evidence of decreasing use for lower economic level with little difference between the low-middle (OR 0.94 c.f. high) and middle (OR 0.95 c.f. high) in South Korea. The pattern of age, sex and economic level was the same as univariable logistic regression in Taiwan.

**Table 1:** Demographic characteristics of western-medicine-only users (WM-only users), traditional-medicine-only users (TM-only users) and those both use WM and TM (WM &TM users) in South Korea and Taiwan in 2011.

		South Korea							Taiwan						
		WM-only users		TM-only users		WM &TM users			WM-only users		TM-only users		WM &TM users		
		(n = 648,168)		(n = 8,763)		(n = 252,715)			(n = 518,808)		(n = 10,547)		(n = 249,982)		
		Number of users	%	Number of users	%	Number of users	%	*P	Number of users	%	Number of users	%	Number of users	%	**P
Sex															
	Male	328,143	50.6	5,751	65.6	103,404	40.9	< .001	271,516	47.7	5,721	54.2	99,180	39.7	< .001
	Female	320,025	49.4	3,012	34.4	149,311	59.1		247,292	52.3	4,826	45.8	150,802	60.3	
Age (years)															
	0–20	180,969	27.9	685	7.8	25,756	10.2	< .001	90,923	17.5	1,515	14.4	36,990	14.8	< .001
	21–40	184,761	28.5	3,875	44.2	62,912	24.9		164,777	31.8	4,668	44.3	94,151	37.7	
	41–60	188,077	29.0	3,732	42.6	100,422	39.7		167,890	32.4	3,847	36.5	83,917	33.6	
	61–80	80,248	12.4	428	4.9	56,501	22.4		78,060	15.1	472	4.5	31,338	12.5	
	>80	14,113	2.2	43	0.5	7,124	2.8		17,158	3.3	45	0.4	3,586	1.4	
Economic level															
	low	22,482	3.5	95	1.1	9,297	3.7	< .001	84,810	16.6	1,653	15.9	33,775	13.7	< .001
	Low-middle	131,046	20.2	1,990	22.7	52,254	20.7		248,170	48.7	4,826	46.5	124,455	50.6	
	middle	165,640	25.6	2,622	29.9	62,182	24.6		107,780	21.1	2,251	21.7	55,273	22.5	
	high	329,000	50.8	4,056	46.3	128,982	51		69,274	13.6	1,643	15.8	32,667	13.3	

*p-value: The Chi squared test was used to examine the relationship between WM-only users, TM-only users and those use TM & WM in South Korea.

** p-value: The Chi squared test was used to examine the relationship between WM-only users, TM-only users and those use TM & WM in Taiwan.

**Table 2:** Odds ratios of traditional medicine use in South Korea and Taiwan in 2011 using univarible and multivariable logistic regression.

	South Korea				Taiwan			
	Univarible	p	Multivariable	p	Univarible	p	Multivariable	p
	OR (95%CI)		AOR (95%CI)		OR (95%CI)		AOR (95%CI)	
Age (years)								
0–20	0.29 (0.28–0.30)	<0.001	0.31 (0.30–0.31)	<0.001	2.00 (1.93–2.08)	<0.001	1.97 (1.90–2.05)	<0.001
21–40	0.71 (0.69–0.73)		0.75 (0.73–0.77)		2.83 (2.73–2.94)		2.77 (2.67–2.87)	
41–60	1.09 (1.06–1.12)		1.15 (1.12–1.19)		2.47 (2.38–2.56)		2.39 (2.30–2.48)	
61–80	1.40 (1.36–1.44)		1.46 (1.42–1.51)		1.93 (1.85–2.00)		1.88 (1.81–1.96)	
>80	1		1		1		1	
Sex								
Male	1	<0.001	1	<0.001	1	<0.001	1	<0.001
Female	1.43 (1.42–1.44)		1.40 (1.39–1.41)		1.63(1.61–1.64)		1.62(1.61–1.64)	
Economic level								
Low	1.03 (1.01–1.06)	<0.001	0.90 (0.87–0.92)	<0.001	0.85(0.84–0.87)	<0.001	0.89(0.87–0.90)	<0.001
Low-middle	1.02 (1.01–1.04)		0.94 (0.93–0.95)		1.05(1.04–1.07)		1.02(1.00–1.03)	0.012
Middle	0.97 (0.96–0.98)		0.95 (0.94–0.96)		1.08(1.06–1.10)		1.02(1.00–1.03)	0.015
High	1		1		1		1	

OR = odds ratio,AOR = adjusted odds ratio,CI = confidence interval. Multivariable logistic regression analysis adjusted for sex, age and economic level.

p-values were determined by the Wald test.

### Utilization patterns of TM visits in South Korea and Taiwan

**Fig 2:**
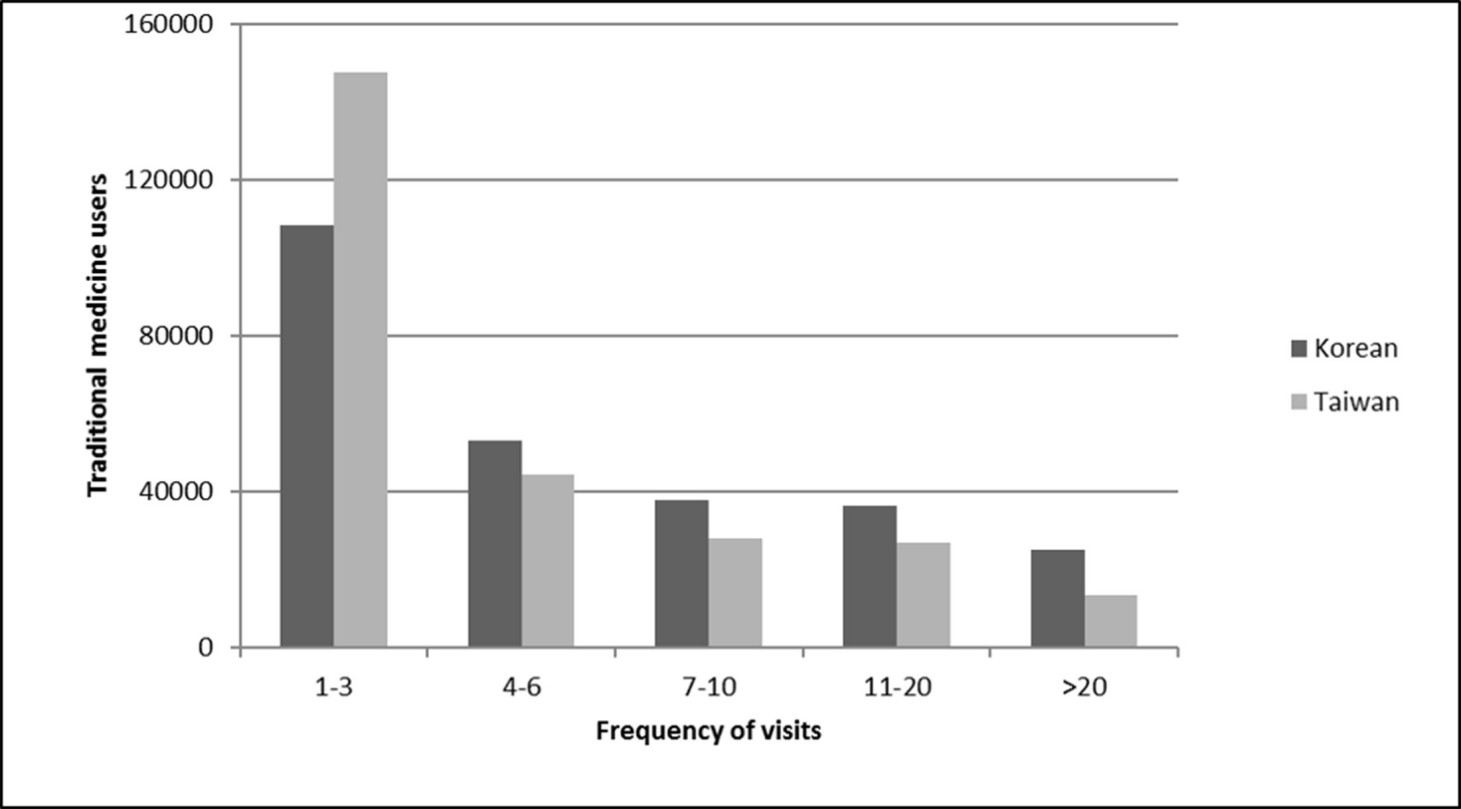
The frequencies of traditional medicine visits in South Korea and Taiwan.

There was a mean of 5.7 ± 7.5 TM visits (median: 3, ranging from 1 to 137) among individuals who used TM services in Taiwan in 2011, which is lower than the mean of 8.9 ± 14.0 TM visits (median: 4, ranging from 1 to 892) in South Korea. The frequencies of the TM visits in both countries are shown in Fig 2. Most people had 1–3 TM visits in one year in both countries. Table 3 describes the patterns of TM use by patients in both countries. Small differences in the distribution of visits by season were observed between the countries, with larger for health care institution type. The seasonal variation in TM visits showed a slight decrease in winter in both countries. Among the service providers, primary care clinics provided the most TM services (Taiwan 91.3% and South Korea 82.4%), followed by hospitals (Taiwan 8.7% and South Korea 1.7%). The ratios of TM hospital visits to TM clinics visits were 1:47.7 in South Korea and 1:10.6 in Taiwan. The number of users in other types of institution was much different in two countries (Taiwan 0.1% and South Korea 27.4%).

**Table 3:** Utilization pattern of traditional medicine visits in South Korea and Taiwan in 2011.

	South Korea			Taiwan				
	Number of visits	%	Number of users	%	Number of visits	%	Number of users	%
**Visit season**								
Spring	598,207	25.7	133,254	25.0	385,430	25.8	129,249	25.1
Summer	596,119	25.6	132,702	24.9	379,277	25.4	126,818	24.6
Fall	580,215	24.9	130,724	24.5	382,659	25.7	126,276	24.5
Winter	553,806	23.8	136,340	25.6	344,378	23.1	132,188	25.7
**Institution types of visits**								
Hospital	40,235	1.7	17,361	4.7	128,660	8.7	23,774	8.8
Clinic	1,919,349	82.4	251,579	67.9	1,357,714	91.3	246,512	91.1
*Other	368,763	15.8	101,701	27.4	1,415	0.1	304	0.1

*Other: include public health center, nursing house and school health center and other place offer the medical services.

### The Frequency of disease categories of TM visits in South Korea and Taiwan

Table 4 and Table 5 shows the disease categories of TM use in South Korea and Taiwan. Among the TM visits in South Korea in 2011, the most common ailments were musculoskeletal system and connective tissue diseases, which accounted for 45.1% of the total TM visits in 2011, followed by diseases of the digestive system (18.2%) and injury, poisoning and certain other consequences of external causes (16.5%). Among the TM visits in Taiwan in 2011, the most common ailments were Symptoms, signs, and ill-defined conditions which accounted for 22.1% of the total TM visits, followed by respiratory system diseases (16.2%) and musculoskeletal system and connective tissue diseases (13.4%).

**Table 4:** Disease diagnosis categories of traditional medicine visits and users in South Korea in 2011.

Disease Diagnosis Categories	ICD-10	Number of visits	%	Number of users	%
Certain infectious and parasitic diseases	A00-B99	2,528	0.1	720	0.2
Neoplasms	C00-D48	4,598	0.2	502	0.1
Diseases of the blood and blood-forming organs and certain disorders involving the immune mechanism	D50-D89	164	0	53	0
Endocrine, nutritional and metabolic diseases	E00-E90	6,677	0.3	1,136	0.2
Mental and behavioural disorders	F00-F99	17,919	0.8	3,884	0.8
Diseases of the nervous system	G00-G99	84,485	3.6	13,963	3.1
Diseases of the eye and adnexa	H00-H59	5,450	0.2	963	0.2
Diseases of the ear and mastoid process	H60-H95	11,706	0.5	1,845	0.4
Diseases of the circulatory system	I00-I99	19,070	0.8	2,058	0.4
Diseases of the respiratory system	J00-J99	57,145	2.5	15,970	3.5
Diseases of the digestive system	K00-K93	423,789	18.2	111,072	24.3
Diseases of the skin and subcutaneous tissue	L00-L99	18,767	0.8	3,501	0.8
Diseases of the musculoskeletal system and connective tissue	M00-M99	1,049,499	45.1	153,056	33.5
Diseases of the genitourinary system	N00-N99	9,617	0.4	2,320	0.5
Pregnancy, childbirth and the puerperium	O00-O99	405	0	183	0
Certain conditions originating in the perinatal period	P00-P96	54	0	22	0
Congenital malformations, deformations and chromosomal abnormalities	Q00-Q99	412	0	96	0
Symptoms, signs and abnormal clinical and laboratory findings, not elsewhere classified	R00-R99	68,818	3	17,766	3.9
Injury, poisoning and certain other consequences of external causes	S00-T98	385,308	16.5	91,053	19.9
Codes for special purposes	U00-U99	155,748	6.7	32,541	7.1
External causes of morbidity and mortality	V01-Y98	50	0	8	0
Factors influencing health status and contact with health services	Z00-Z99	6,138	0.3	4,799	1

**Table 5:** Disease diagnosis categories of traditional medicine visits and users in Taiwan in 2011.

Disease Diagnosis Categories	ICD-9	Number of visits	%	Number of users	%
Infectious and parasitic diseases	001–139	4,883	0.3	2,360	0.5
Neoplasms	140–239	11,855	0.8	1,716	0.4
Endocrine, nutritional and metabolic diseases, and immunity disorders	240–279	22,587	1.5	5,599	1.2
Diseases of the blood and blood-forming organs	280–289	4,576	0.3	1,524	0.3
Mental disorders	290–319	13,048	0.9	3,991	0.9
Diseases of the nervous system and sense organs	320–389	43,140	2.9	14,487	3.1
Diseases of the circulatory system	390–459	39,255	2.6	7,839	1.7
Diseases of the respiratory system	460–519	241,078	16.2	75,292	16.2
Diseases of the digestive system	520–579	184,524	12.4	53,077	11.4
Diseases of the genitourinary system	580–629	127,056	8.5	33,170	7.1
Complications of pregnancy, childbirth, and the puerperium	630–679	1,809	0.1	768	0.2
Diseases of the skin and subcutaneous tissue	680–709	66,924	4.5	19,902	4.3
Diseases of the musculoskeletal system and connective tissue	710–739	200,110	13.4	71,444	15.4
Congenital anomalies	740–759	2,108	0.1	689	0.1
Certain conditions originating in the perinatal period	760–779	37	0.0	13	0.0
Symptoms, signs, and ill-defined conditions	780–799	330,103	22.1	90,991	19.6
Injury and poisoning	800–999	198,545	13.3	81,406	17.5
External causes of injury and supplemental classification	V01-E999	106	0.0	77	0.0

### Utilization patterns of traditional medical treatments and examinations

Table 6 shows the prevalence of specific TM treatments used in South Korea and Taiwan. In South Korea, acupuncture accounted for 59.7%, followed by cupping (12.8%), moxibustion (7.6%), electro-acupuncture (5.2%), and hot/cold pack therapy (3.6%). In Taiwan, acupuncture and moxibustion accounted for 30.5% of the treatments. Powdered herbal preparations comprised only 5.2% of the treatments in South Korea, whereas in Taiwan, 61.2% of the patients were treated with these herbal preparations. Both cupping and hyperthermia therapy were used only in South Korea, whereas manipulative therapy was used only in Taiwan (6.9%). Furthermore, pulse diagnosis and tongue diagnosis instruments were used in Taiwan, and pulse diagnosis, meridian diagnosis, and Yangdorak (Ryodoraku) were used in South Korea.

**Table 6:** Treatment and examination types among traditional medicine visits in South Korea and Taiwan in 2011.

	South Korea		Taiwan	
	Visits	%	Visits	%
Treatment type				
Acupuncture	3,683,036	59.7	623,193	30.5
Powered herbal preparation	683,338	11.1	1,250,717	61.2
Electro-acupuncture	321,434	5.2	28,825	1.4
Manipulative therapy	*NA	*NA	141,607	6.9
Cupping	788,671	12.8	*NA	*NA
**Moxibustion	471,264	7.6	*NA	*NA
Hot/cold pack	220,034	3.6	*NA	*NA
Examination type				
Pulse diagnosis	2,570	51.8	66	29.7
Tongue diagnosis	*NA	*NA	156	70.3
Meridian diagnosis	1,157	23.3	*NA	*NA
Yangdorak(Ryodoraku)	1,235	24.9	*NA	*NA

*NA: Items were not covered by national health insurance and data were not available.

** Moxibustion was reimbursed together with acupuncture treatment in Taiwan.

## Discussion

### Demographic characteristics of health care institution users

Private TM clinics in South Korea and Taiwan outnumber those in other countries where TM is being practiced. According to the National Statistical Bureau, 16 TM hospitals and 3,411 TM clinics were located across Taiwan in 2011 [11]. In South Korea, there were 168 TM hospitals and 12,061 TM clinics in the same year [6]. In the present study, the ratio of patients who visited TM hospitals to patients who visited TM clinics were approximately 1:10 in Taiwan and 1:47 in South Korea, implying that people visited TM clinics more than TM hospitals when seeking TM treatment in both countries. Compared to the ratio of TM hospitals/clinics (1:213 in Taiwan and 1:72 in South Korea), there were more TM hospital visits in Taiwan than in South Korea.

Taiwan and South Korea have similar NHI systems. Both are single-payer systems and provide universal coverage. Furthermore, most WM and TM hospitals and WM and TM clinics collaborate with the NHI system in both countries. Outpatient visits of TM services either in primary care clinics or in hospitals are reimbursed by the NHI of South Korea and Taiwan. However, inpatient TM services in hospital were reimbursed only in South Korea, but not in Taiwan.

The dual WM and TM health care systems have a long history in both Taiwan and South Korea. Although WM is the leading official health care system at present, the statuses of WM doctors and TM doctors are similar in both countries [3]. Most people in both countries perceive WM as having faster effects and with a well-founded evidence base, whereas TM is thought to treat patients as a whole person by immediately treating not only the disease itself but also adjusting to the whole-body situation [12]. Thus, combining the strengths of WM and TM through integrative use might be more in line with patient preferences. Some hospitals have established joint WM and TM treatment centers. Unfortunately, medical insurance did not cover both WM and TM treatment on the same day for the same disease in South Korea until recently. In Taiwan, no strict provisions existed that prohibit a combined WM and TM treatment on the same day. Furthermore, to promote combined treatment, extra subsidies are available from the NHI when a patient uses combined WM and TM treatment for cancer and cerebrovascular diseases in Taiwan [13].

In terms of the distribution of the utilization of TM services in this study using multivariable logistic regression, the number of TM users increased with age in South Korea; in Taiwan, the prevalence of TM use increased from age 0–20 to age 21–40, and then decreased for older age groups. The results were consistent with the Korean Health Panel results from 2008 to 2009, and with another previous study from Taiwan [14]. In order to elucidate the effect of age, sex and economic level on TM use, we have performed three different logistic regression models. When we only included sex and economic level in the multivariable model, we found the direction of the effect of both variables were consistent with univariable analysis. However, if we stratified by sex further, we found the effects of economic level between male and female were in opposite direction. Thus, we speculated that sex is a modifying factor between economic level and TM use. When we included age and economic level in the model, we found the effects of the economic level were changed. Thus, we speculated that age may have modifying or confounding effects. We stratified by age further (data not shown), and found that age is also a modifying factor. According to the aforementioned results, we speculated that sex and age are effect modifiers between economic level and TM use.

### Disease categories for TM visits in South Korea and Taiwan

Approximately half of the TM visitors in South Korea were classified in the musculoskeletal disease category (45.1%), followed by the digestive disease category (18.2%). A previous study indicated that more than half of the TM visits were for diagnoses of musculoskeletal system disorders in South Korea [3]. This is consistent with our study results. Compared with South Korea, the common disease categories for TM visits were not as concentrated in Taiwan. Symptoms, signs, and ill-defined conditions (22.1%), respiratory system diseases (16.2%) and musculoskeletal system and connective tissue disorders (13.4%) were ranked the top three most common diseases for which TM treatment was sought in Taiwan. The first nationwide survey of TM visits published in 2007 indicated that respiratory diseases represented the most common disease category for TM visits from 1996 to 2001 [14]. According to a previous study, which focused on a specific age group, the most common reason for TM visits in children was also respiratory diseases [15]. Yang et al. [16] have also shown that the most common reason for TM visits was musculoskeletal system diseases in elderly patients in Taiwan.

Acupuncture was the most common TM treatment type in South Korea. Acupuncture has been shown to have effects on relieving chronic pain and improving the conditions of musculoskeletal disorders [14]. The differences of common types of treatment used by TM doctors between the two countries (acupuncture in South Korea and powered herbal preparation in Taiwan) may be due to the differences of diseases diagnoses commonly treated in both countries. There were significantly more patients with musculoskeletal system disorders seeking TM service in South Korea than in Taiwan.

South Korea and Taiwan have similar health care systems. Both the WM and TM health care systems are reimbursed by the NHI. Due to the differences of diseases commonly treated in TM in the two countries, there were limitations when performing a comparative study. Moreover, TM doctors need to use the ICD system that is designed for WM for insurance reimbursement. According to the ICD-11 beta draft, the newly designed ICD-11 includes a TM diagnosis section. The TM diagnosis section was established due to TM use in China, South Korea and Japan. The limitations of the disease systems classification may be improved when the ICD-11 system is put into practice [17].

### Differences in reimbursed treatment and examination items by the NHI for TM treatment between the two countries

TM treatment and TM examination are reimbursed by NHI both in South Korea and Taiwan. Acupuncture, moxibustion, powered herbal preparation, electro-acupuncture and pulse diagnosis were all reimbursed in both countries. Cupping, hot/cold packs, meridian diagnosis and Yangdorak were only reimbursed in South Korea. However, manipulative therapy and tongue diagnoses were only reimbursed in Taiwan.

The most obvious differences in commonly used TM treatments in these two countries were in the most common procedures: acupuncture in South Korea and powdered herbal preparations in Taiwan. We also found that the rate of powdered herbal preparations use was much higher in Taiwan, perhaps because most TM doctors in Taiwan use powered herbal preparation for their primary treatment type rather than acupuncture, and for Korean TM doctors, the opposite is true. There were only 58 types of herbal formulas that were eligible for reimbursement in Korean NHI, but there were 337 types in Taiwan. Korean TM doctors might feel limited in their use of powered herbal preparations, and therefore, most of them choose to use acupuncture as their primary treatment type.

One of the important policies raised in the 3rd Foster Development Plan of Korean Medicine (2016–2020) was the expansion of the coverage range of Korean medicines in the NHI system. This policy called on scholars to perform more international comparative studies and evidence-based medicine studies to establish a more powerful background for the safety and effectiveness of Korean medicine [18]. Our prediction is that some changes will need to be made in the utilization of TM in South Korea in the coming years. Further research is also needed to follow up the clinical effectiveness of powdered herbal preparation use in both countries.

Among the countries where TM is a common part of the health system, South Korea and Taiwan are the only two countries that have released most of their NHI data for research studies. To date, nationwide studies have used the NHI database of a single country. This study was the first to present TM use in two countries using nationwide health insurance data. Our results provide a rough outline of the utilization patterns of TM. We believe that future studies can be performed using the framework of our study, while providing more detailed comparisons (e.g., changes in the process of the reimbursement system or the pattern of powered herbal preparation use) between the two countries. More comparative studies between different countries can be performed to discuss the use of TM among different races or ethnic groups. We believe that if the TM treatment can be proven to be beneficial in two or more races or ethnic groups through comprehensive NHI dataset, the results would be more convincing than those of an analysis performed in only one country.

### Strengths and limitations of this study

To the best of our knowledge, this is the first cross-national study of utilization patterns of traditional medicine. Although our study has strength of large scale size and national population-based study, there are still some limitations in this study. First, the use of crude herbal drugs, an important TM treatment, could not be presented for both countries because they are not covered by the NHI. Second, because the insurance coverage was based on the use of the ICD diagnoses, we were forced to use the same system to classify patients in this study; however, the primary dialectic terms used by the TM doctors were the eight principles syndrome differentiation. Third, to maintain consistency between the two NHI databases in each country, we could only extract one year (2011) of data from the whole cohort database for the analysis. Beside, the data we used in this study was over six years old. However, there were no considerable policy changes in the NHI system in either country in recent years. Therefore, our data can still reflect the current situation. Fourth, except sex, age and economic level, there might be still some potential confounders such as education level and occupational status which were not included in this study, due to by lack of this information in the original NHI database. Detailed studies should be conducted by connecting the NHI database with other database such as The Korean Medical Use and Consumption Survey or National Health Pannel. Another limitation is that this study was conducted in two different countries, since the regulations of NHIRD could not allow the database to be transferred to other places. We could only analyze the data separately, and therefore restrictions remained for evaluating the country effect on TM use. For these reasons, this study may not have presented the entire pictures regarding differences in the use of TM services between the two countries.

## Conclusion

Among the numerous countries that use TM, only South Korea and Taiwan provide detailed NHI data for academic purposes. According to the NHI database, about one fourth of the NHI beneficiaries of South Korea and Taiwan had TM use in 2011. Different TM utilization patterns existed between South Korea and Taiwan, which might be due to the differences in insurance coverage of the two countries.
